# P05.63. Perspectives of breast cancer survivors about participating in an acupuncture clinical trial for hot flashes: a qualitative study

**DOI:** 10.1186/1472-6882-12-S1-P423

**Published:** 2012-06-12

**Authors:** C Seluzicki, E Mackenzie, F Barg, R Leed, M Bowman, J Mao

**Affiliations:** 1 University of Pennsylvania Health System, Philadelphia, USA; 2 Oregon Health Sciences University, Portland, USA

## Purpose

Evidence-based decisions about acupuncture use rests on outcomes of randomized clinical trials, but little is known about cancer patients’ attitudes and beliefs about participating in clinical trials involving acupuncture. We conducted this study to understand how breast cancer survivors make decisions regarding participation in an acupuncture clinical trial for hot flashes.

## Methods

Twenty-five breast cancer survivors (12 African-Americans/13 Caucasian) were interviewed and verbatim transcripts were made. These transcripts were analyzed with NVivo software, and major recurring themes were identified. Multiple readers independently verified the results.

## Results

Five major themes emerged: (1) symptom appraisal (e.g. determining if/when symptoms become bothersome enough to necessitate intervention), (2) practical barriers (e.g. distance, travel), (3) beliefs about the interventions (e.g. fear of needles, dislike of medications), (4) comfort with clinical trials (e.g. randomization, blinding, placebo), (5) trust and altruism. Breast cancer survivors weighed benefits and costs associated with the decision to participate in a clinical trial involving acupuncture. Symptom appraisal was weighed against practical barriers to determine whether the potential benefits outweighed the costs (in time and effort) involved. Women also reflected on the nature of the interventions. Some favored acupuncture due to concerns about taking additional medications; others favored medication due to their fear of needles and/or skepticism about acupuncture’s effectiveness. Finally, women were more likely to express willingness to participate in a clinical trial if they had some understanding of the purpose of trial design (e.g. randomization, placebo, blinding); suspicions about placebo, blinding and randomization were barriers to participation. However, the presence of trust and altruism mitigated these concerns.

## Conclusion

Breast cancer survivors expressed specific attitudes about perceived barriers and facilitators to participating in acupuncture research. Incorporating patients’ perspectives in study design and recruitment strategies may facilitate conducting rigorous clinical trials of acupuncture to guide evidence-based care.

